# Dynamics of transcriptome changes during subcutaneous preadipocyte differentiation in ducks

**DOI:** 10.1186/s12864-019-6055-9

**Published:** 2019-09-02

**Authors:** Zheng Wang, Zhong-Tao Yin, Fan Zhang, Xiao-Qin Li, Si-Rui Chen, Ning Yang, Tom E. Porter, Zhuocheng Hou

**Affiliations:** 1 National Engineering Laboratory for Animal Breeding and Key Laboratory of Animal Genetics, Breeding and Reproduction, MARA; Department of Animal Genetics and Breeding, China Agricultural University, Beijing, 100193 China; 2 Department of Animal and Avian Sciences, University of Maryland, College Park, MD 20742 USA

**Keywords:** Pekin duck, Subcutaneous fat, Preadipocyte differentiation, Transcriptome, Transcription factors, Regulation network model

## Abstract

**Background:**

Pekin duck is an important animal model for its ability for fat synthesis and deposition. However, transcriptional dynamic regulation of adipose differentiation driven by complex signal cascades remains largely unexplored in this model. This study aimed to explore adipogenic transcriptional dynamics before (proliferation) and after (differentiation) initial preadipocyte differentiation in ducks.

**Results:**

Exogenous oleic acid alone successfully induced duck subcutaneous preadipocyte differentiation. We explored 36 mRNA-seq libraries in order to study transcriptome dynamics during proliferation and differentiation processes at 6 time points. Using robust statistical analysis, we identified 845, 652, 359, 2401 and 1933 genes differentially expressed between -48 h and 0 h, 0 h and 12 h, 12 h and 24 h, 24 h and 48 h, 48 h and 72 h, respectively (FDR < 0.05, FC > 1.5). At the proliferation stage, proliferation related pathways and basic cellular and metabolic processes were inhibited, while regulatory factors that initiate differentiation enter the ready-to-activate state, which provides a precondition for initiating adipose differentiation. According to weighted gene co-expression network analysis, pathways positively related to adipogenic differentiation are significantly activated at the differentiation stage, while WNT, FOXO and other pathways that inhibit preadipocyte differentiation are negatively regulated. Moreover, we identified and classified more than 100 transcription factors that showed significant changes during differentiation, and found novel transcription factors that were not reported to be related to preadipoctye differentiation. Finally, we manually assembled a proposed regulation network model of subcutaneous preadipocyte differentiation base on the expression data, and suggested that *E2F1* may serve as an important link between the processes of duck subcutaneous preadipocyte proliferation and differentiation.

**Conclusions:**

For the first time we comprehensively analyzed the transcriptome dynamics of duck subcutaneous preadipocyte proliferation and differentiation. The current study provides a solid basis for understanding the synthesis and deposition of subcutaneous fat in ducks. Furthermore, the information generated will allow future investigations of specific genes involved in particular stages of duck adipogenesis.

**Electronic supplementary material:**

The online version of this article (10.1186/s12864-019-6055-9) contains supplementary material, which is available to authorized users.

## Background

Adipose tissue has multiple roles in the regulation of insulin sensitivity [1], feed conversion ratio [2] and meat quality [3, 4] in animals. Certain amounts of intramuscular fat are required to meet consumer needs. Most investigations have focused on mammals, specifically the mouse and human. Adipocyte differentiation is a complex process regulated by multiple transcription factors (TFs), which affect expression level and activity of hundreds of proteins, resulting in dramatic changes in phenotypes [5–7]. Peroxisome Proliferator Activated Receptor Gamma (*PPARγ*) and CCAAT Enhancer Binding Protein Alpha (*C/EBPα*) are two master TFs supported by overwhelming evidence in vivo and in vitro [6],and many TFs and signaling pathways also participate in adipogenesis progression, playing either a positive or negative role [6, 8–10]. Gene networks, which integrate the mRNA and microRNA data, of brown adipose tissue have been created in the most recent study in the mouse model [11]. Avian species do not have brown adipose tissue (BAT), and lack Uncoupling Protein 1 (*UCP1*) [12]. Birds were thought to only share white adipose tissue [13, 14]. The subcutaneous white adipose tissue has important beneficial characteristics, including storage of lipid, secretion of adipokines, and anti-inflammatory roles [15]. Most studies on preadipocyte proliferation and adipogenic differentiation have been performed in vitro using human and murine cell lines [16, 17]. Recent studies showed that the process of preadipocyte differentiation in chicken has both similarities and differences with mammals [18]. Very limited studies based on candidate genes showed that several well-known TFs have similar expression patterns in chickens [19] and ducks [20] during adipocyte differentiation.

Duck is one of the most important meat sources in Asia, especially in China [21]. Roast Pekin duck requires a considerable skin-fat content, and Pekin duck has been used as a new model for studying behavior [22], meat quality [23], growth [4, 24] and fat synthesis and deposition [25]. Understanding the genetics of adipocyte differentiation is critical for controlling adipocyte deposition in ducks. However, no transcriptomic data have been reported during duck adipocyte differentiation. Elucidating the adipocyte differentiation process at the transcriptional level would be the foundation for further understanding of adipocyte biology in ducks.

This study aims to comprehensively analyze and compare gene expression profiles of 6 different time points during subcutaneous preadipocyte proliferation and differentiation. Our study explored 36 mRNA-seq libraries to obtain high quality differentially expressed gene sets across time points and constructed co-expression gene networks. We provide a reliable set of differentially expressed genes (DEGs) representing preadipocyte proliferation and differentiation. Furthermore, we have identified many known and novel TFs and signaling pathways associated with duck preadipocyte proliferation and differentiation. Finally, we provide a proposed regulation network model of subcutaneous preadipocyte differentiation.

## Results

### Duck subcutaneous preadipocyte differentiation

**Fig. 1:**
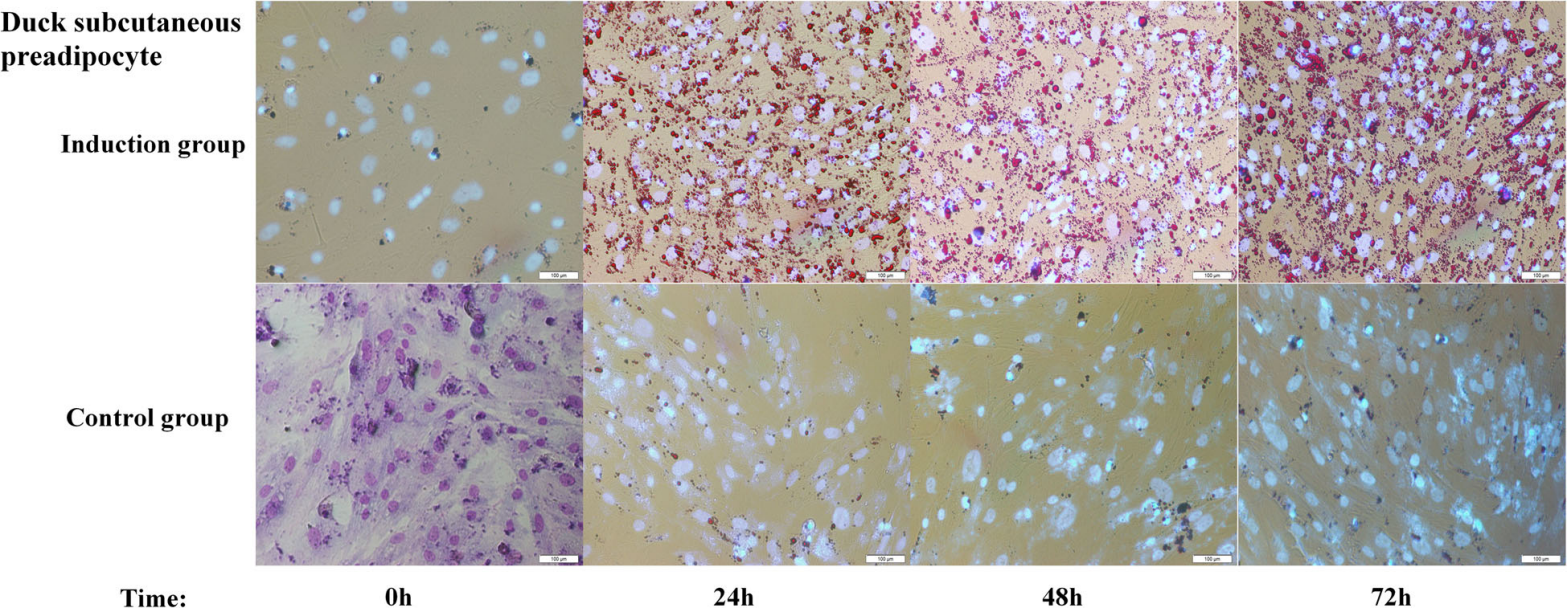
Morphological changes of duck subcutaneous preadipocyte cultured in differentiation medium (Induction group) or growth medium (Control group) at 200×. The picture in the lower left corner is the cell diagram after staining with Giemsa at 0 h. Bar, 100 μm

**Fig. 2:**
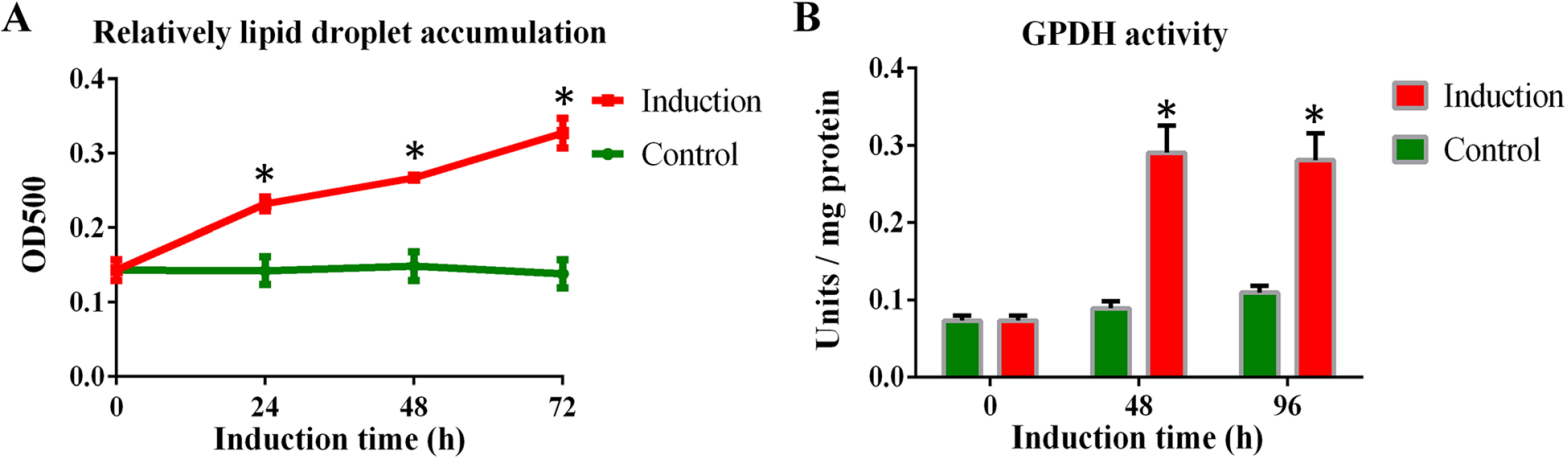
Intracellular lipid droplet accumulation and glycerol-3-phosphate dehydrogenase (GPDH) activity in duck preadipocytes cultured in differentiation medium (Induction) or growth medium (Control). (**a**) Relative quantification of lipid droplet accumulation within 72 h after induction. (**b**) GPDH activities were analyzed at 0 h, 48 h and 96 h post-induction. Bars indicate SD of the mean values (*n* = 3). *Statistically significant differences compared to the levels at 0 h (*P* < 0.05)

Cell morphological characteristics were measured at 0 h, 24 h, 48 h and 72 h during preadipocyte differentiation. Duck subcutaneous preadipocyte cultured in differentiation medium containing 300 μM oleic acid, showed a remarkable increase in lipid deposition compared with those cultured in the growth medium without oleic acid (Fig. 1). Lipid droplets in cells form as early as 24 h and showed a gradual increase in the percentage of cells with increased intracellular lipid content. Similarly, preadipocyte cultured in the induction medium showed significantly higher accumulation of lipid droplets compared with the control medium (Fig. 2a). GPDH enzyme activity increased significantly in comparison with control group at 48 h and 96 h of differentiation (Fig. 2b), with the addition of oleic acid. These results suggest that oleic acid alone can successfully induce differentiation in duck preadipocyte.

### Transcriptome dynamics during preadipocyte proliferation and differentiation

Gene expression was studied over 120 h in a total of 36 samples (6 biological replicates at each point for each condition) using mRNA-seq. Each mRNA-seq library was sequenced to more than 20 million reads. Subsequently, clean reads were uniquely mapped to the duck reference genome (*Anas_platyrhynchos*.BGI_duck_1.0) with statistics for the mRNA-seq data referred to in Additional file 1: Table S1. All samples were hierarchically clustered based on Spearman’s correlation of gene expression. If the sample could not be clustered with other samples from the same time point, the sample was considered an outlier and was excluded from further analysis. To represent count data variability, standard error values were calculated per gene based on biological replicates FPKM (*n* = 5–6) at each time point, with the exception of the reference genes, where SE were calculated based on all samples and across all experimental groups (excluding the outlier sample -48 h-1 and 0 h-5, *n* = 34) (Additional file 2: Figure S1). The remaining 34 samples were used for subsequent analysis, and FPKM of obtained transcripts are provided in Additional file 3: Table S2.

**Fig. 3:**
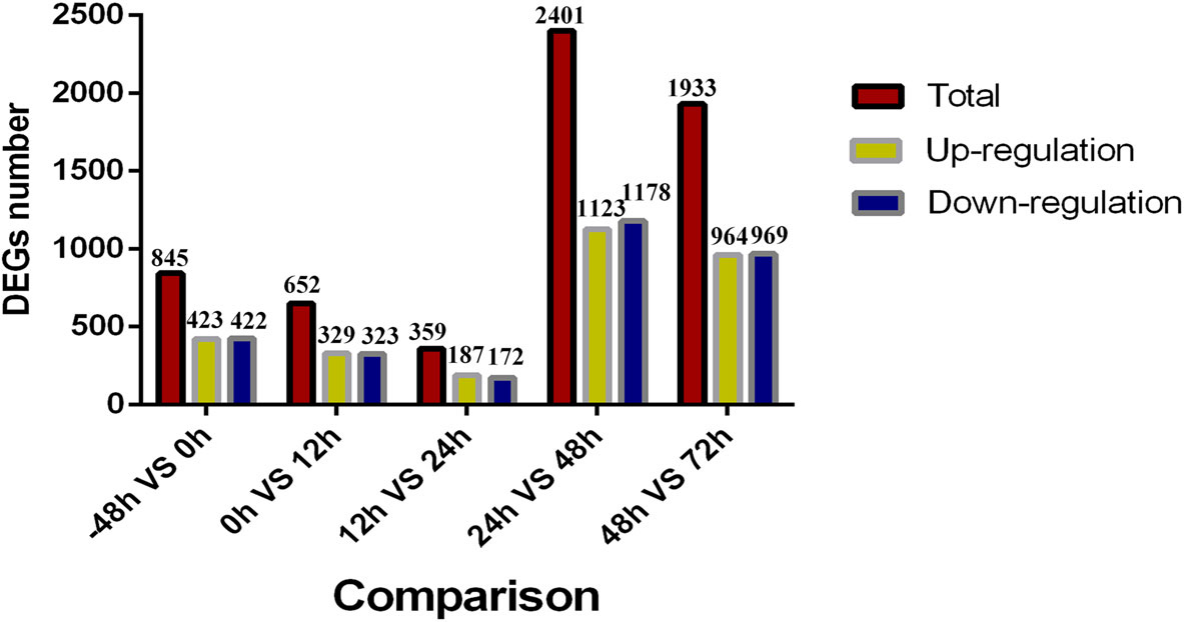
Histogram of the number of DEGs at different time points during preadipocyte differentiation stages

Using a robust statistical analysis, we identified 845, 652, 359, 2401 and 1933 genes showing differential expression between -48 h and 0 h, 0 h and 12 h, 12 h and 24 h, 24 h and 48 h, 48 h and 72 h, respectively (FDR < 0.05, FC > 1.5) (Fig. 3). We found that gene expression patterns changed substantially between 24 h and 48 h in terms of the most DEGs at this stage relative to all other comparisons. A complete list of DEGs seen during the differentiation process is provided in Additional file 4: Table S3.

### Functional analysis of differentially expressed genes during preadipocyte proliferation

Transcriptome expression analyses of duck preadipocyte proliferation stages showed that 423 genes were over-expressed at -48 h, and 422 genes were over-expressed at 0 h from 845 DEGs (− 48 h compared to 0 h). GO enrichment analysis showed genes over-expressed at -48 h compared to 0 h include those involved in mRNA catabolic process, mitotic cell cycle phase transition and cholesterol biosynthetic process. Genes over-expressed at 0 h compared to -48 h include those involved in actin cytoskeleton organization, calcium ion binding and collagen fibril organization. Surprisingly, 26 and 22 focal adhesion terms were significantly enriched at each time point (Table 1; Full list of enriched GO terms. Also refer to Additional file 5: Table S4). Moreover, KEGG pathway analysis showed genes over-expressed at -48 h compared to 0 h include those involved in ribosome, cell cycle and glycolysis. Genes over-expressed at 0 h compared to -48 h include those involved in regulation of ECM-receptor interaction, actin cytoskeleton and MAPK signaling pathway (Table 1; Full list of enriched pathways categories. Also refer to Additional file 6: Table S5).

**Table 1:** Enriched GO terms and KEGG pathways of DEGs in the proliferation stage

Proliferation stage	GO enrichment	Gene count	Log (q-value)	KEGG pathway	Gene count	Log (q-value)
Up at -48 h	mRNA catabolic process	44	− 23.15	Ribosome	30	−22.09
	Mitotic cell cycle phase transition	45	−16.92	PI3K-Akt signaling pathway	23	−6.36
	Ribosomal subunit	34	−22.87	Cell cycle	20	−12.60
	RNA splicing	29	−8.00	Spliceosome	14	−6.20
	Focal adhesion	26	−7.40	DNA replication	14	−3.14
	Cholesterol biosynthetic process	16	−11.48	Glycolysis	6	−4.953
Up at 0 h	Actin cytoskeleton organization	44	−12.86	Focal adhesion	22	−9.80
	Calcium ion binding	42	−10.43	ECM-receptor interaction	17	−11.44
	Cadherin binding	29	−11.76	Regulation of actin cytoskeleton	12	−2.33
	Focal adhesion	25	−5.88	Calcium signaling pathway	11	−2.24
	Collagen fibril organization	13	−9.30	MAPK signaling pathway	10	−0.86

### Co-expression network and module construction during preadipocyte differentiation

**Fig. 4:**
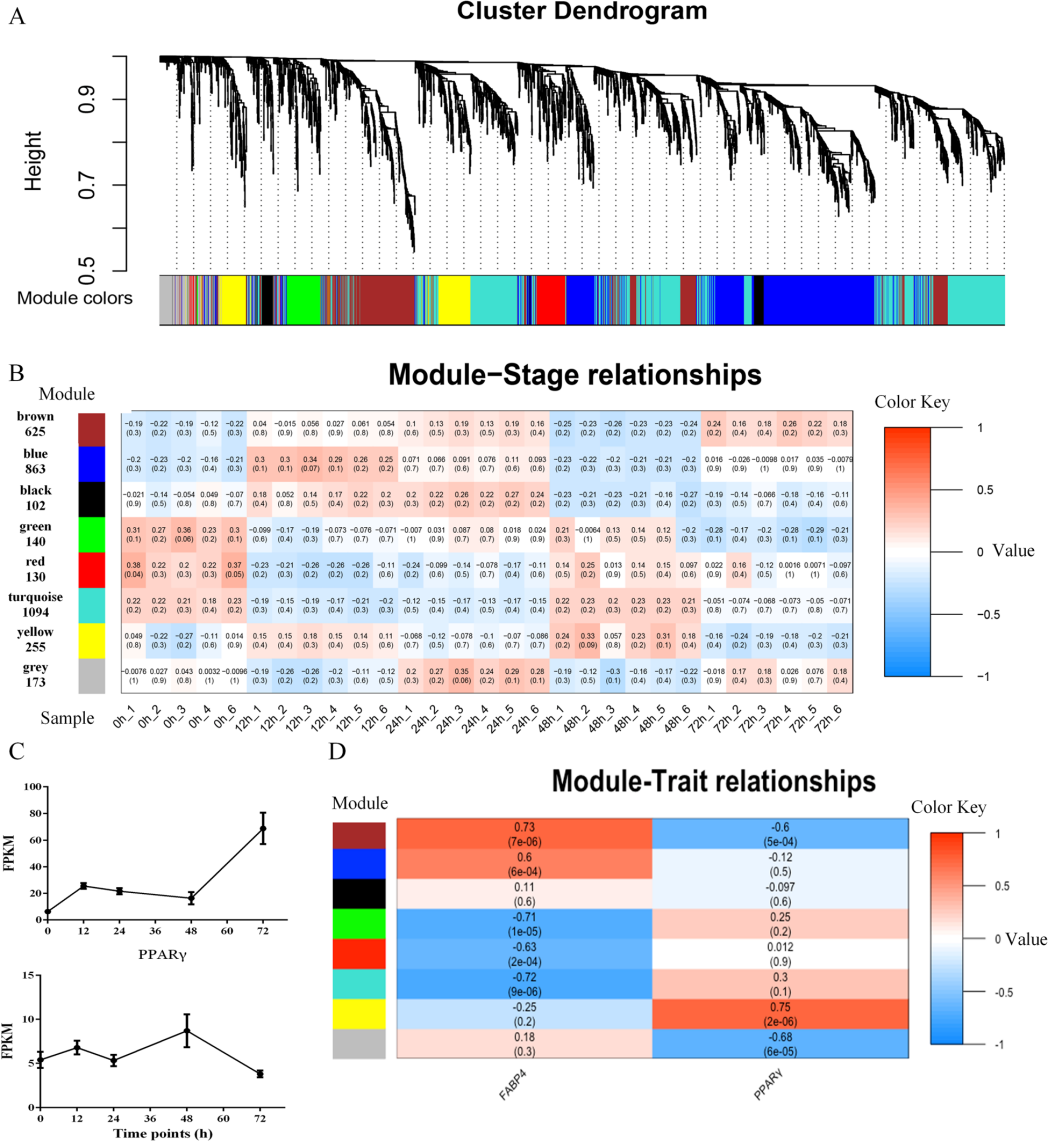
Weighted gene co-expression network analyses (WGCNA) of DEGs identified from differentiation stages. (**a**) Hierarchical cluster tree showing 8 modules of co-expressed genes. Each of the 3382 DEGs are represented by a tree leaf and each of the modules by a major tree branch. The lower panel shows modules in designated colors. (**b**) Module–sample correlations and corresponding *p*-values (in parentheses). The left panel shows the 8 modules and the number of member genes. The color scale on the right shows module–sample correlations from − 1 (blue) to 1 (red). The labels on the bottom panel represent samples at different points in time. (**c**) mRNA-seq expression patterns of *PPARγ* and *FABP4*. (**d**) Module–trait correlations and corresponding p-values (in parentheses). The left panel shows the 8 modules and the colour scale on the right shows module–trait correlations from − 1 (blue) to 1 (red). The left panel “*PPARγ*” (bottom) represents the expression changes of *PPARγ*, which is the key transcription factor activating adipogenic differentiation. The right panel “*FABP4*” (bottom) represents the expression changes of *FABP4*, which is important for lipid hydrolysis and transportation of intracellular free fatty acid

To gain insight into whole gene interaction networks of preadipocyte differentiation and lipid biosynthesis, we performed a Weighted Correlation Network Analysis (WGCNA) to identify groups of co-expressed genes using non-redundant DEGs (*n* = 3382) between any two adjacent time points during the differentiation stages. Modules associated with specific differentiation stage were identified based on the correlation between module eigengene and samples. As shown in the dendrogram (Fig. 4a), 8 consensus modules were identified in the analysis, labeled by different colors, with each containing at least 100 genes (Fig. 4b; Additional file 7: Table S6). MEblue, MEyellow and MEblack modules were highly and specifically accumulated at12h, 24 h and 48 h after differentiation, respectively (Figure 4b; Additional file 8: Figure S2), which indicated that these groups of genes might be responsible for positive regulation during differentiation. In contrast, MEturquoise, MEred and MEgreen modules were highly and specifically accumulated at 0 h and significantly decreased after differentiation (Fig. 4b), indicating that these groups of genes might be involved in maintaining pluripotency of preadipocyte or negative regulation of preadipocyte differentiation. Additionally, the MEbrown module was highly and specifically accumulated at 72 h after differentiation, which indicated that this group of genes might be responsible for lipid deposition of adipose cells at the end of differentiation. A further module-trait relationship analysis, using the expression level of *PPARγ* and Fatty acid binding protein 4 (*FABP4*) as the trait data, revealed that expression patterns of *FABP4* were not only highly correlated with the MEbrown module, but also positively correlated with MEblue and MEyellow modules. *PPARγ* was only correlated with the MEblack module, which might relate to the slow rise of its expression after the beginning of differentiation (Fig. 4c, d).

### Functional analysis of differentially expressed genes during preadipocyte differentiation

According to the above analysis, the expression level of genes belong to the MEblue, MEyellow and MEblack modules increased significantly after induction, but decreased rapidly in late differentiation, so they were considered as early positive response genes of differentiation. The genes in the MEturquosie, MEgreen and MEred modules were considered as early negative response genes, while the genes in the MEbrown module designated late response genes.

In the early positive response genes, most of the enriched GO terms related to the regulation of cellular protein localization, ribosome biogenesis and cellular response to lipid (Table 2; Additional file 5: Table S4). To obtain more detailed information, a pathway analysis was carried out using Metascape. Some primary adipose differentiation-related pathways, including those involved in cell cycle, MAPK, and PI3K-Akt signaling pathway were observed in the early positive response genes (Table 2; Additional file 6: Table S5). The GO enrichment analyses of the early negative response genes revealed distinct enrichment patterns. The three primary enriched terms were the actin filament-based process, response to growth factor, and regulation of system processes (Table 2; Additional file 5: Table S4). Pathway analysis of early negative response genes showed that apoptosis, WNT, FOXO and HIF signaling pathways (which inhibit adipose differentiation) were significantly enriched (Table 2; Additional file 6: Table S5). The functional annotations of the late response genes were linked to cell division, lipid biosynthetic process, steroid biosynthetic process, and fatty acid metabolic processes (Table 2; Additional file 5: Table S4). For pathway analysis, cell cycle, steroid biosynthesis and *PPARγ* signaling pathway were also significantly enriched (Table 2; Additional file 6: Table S5).

**Table 2:** Enriched GO terms and KEGG pathways of different response gene sets at differentiation stages

Differentiation stage	GO enrichment (biological process)	Gene count	Log (q-value)	KEGG pathway	Gene count	Log (q-value)
Early positive response gene set	Muscle structure development	59	−6.72	PI3K-Akt signaling pathway	31	−2.40
	ncRNA metabolic process	52	−6.68	Regulation of actin cytoskeleton	19	−1.39
	Cellular protein localization	46	−5.57	MAPK signaling pathway	17	−0.43
	Ribosome biogenesis	34	−6.12	DNA replication	14	−4.43
Early negative response gene set	Actin filament-based process	63	−6.70	WNT signaling pathway	27	−7.21
	Response to growth factor	57	−5.08	Apoptosis	18	−2.86
	Muscle structure development	52	−4.71	FOXO signaling pathway	17	−2.74
	Regulation of system process	50	−5.51	HIF-1 signaling pathway	14	−2.47
Late response gene set	Cell division	65	−28.52	Cell cycle	18	−7.29
	Lipid biosynthetic process	31	−3.473	Steroid biosynthesis	8	−6.08
	Steroid biosynthetic process	21	−8.212	p53 signaling pathway	8	−2.12
	Fatty acid metabolic process	14	−0.713	PPARγ signaling pathway	7	−1.38

### Expression pattern analysis of differentially expressed TFs during differentiation

**Fig. 5:**
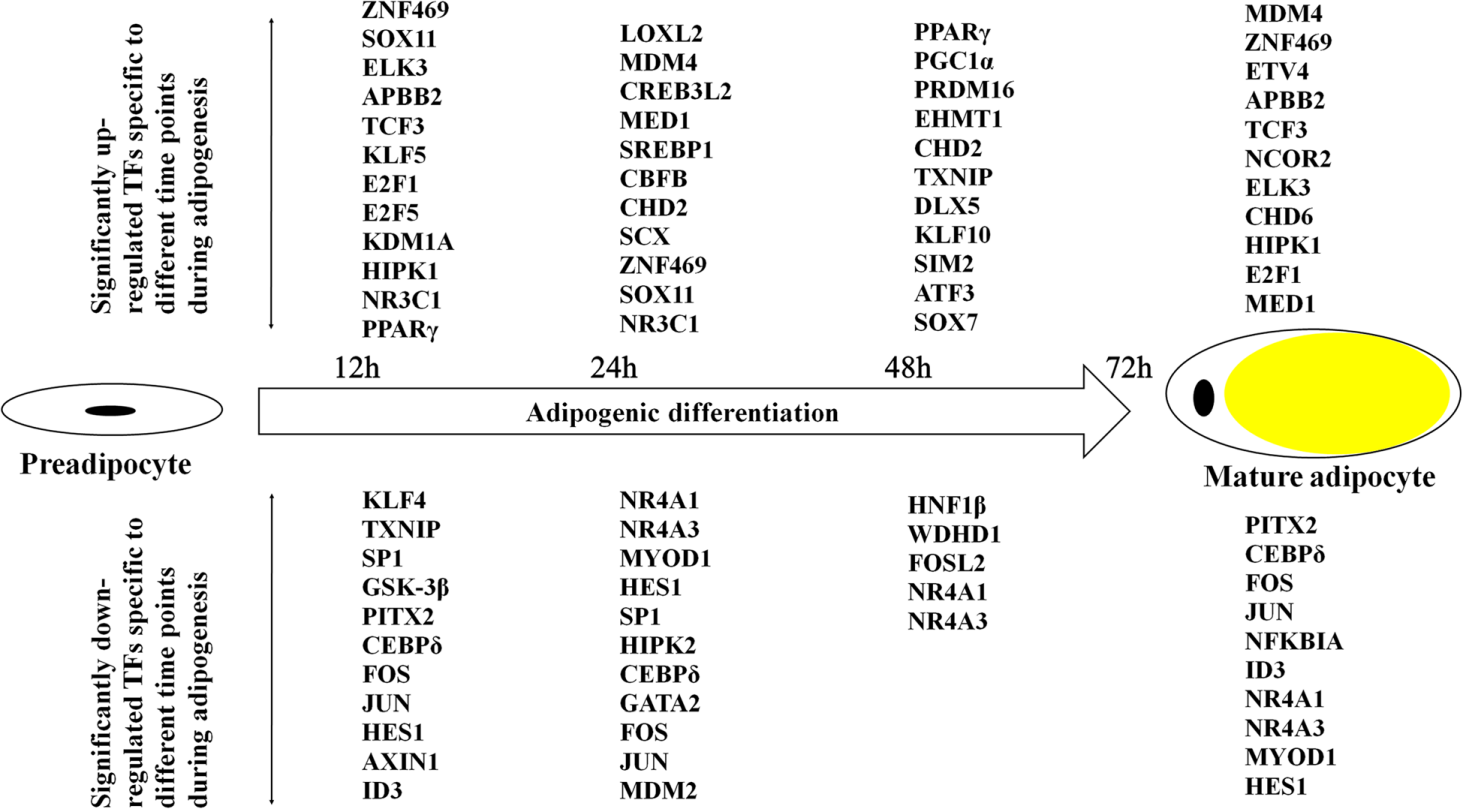
TFs expression at different time points during preadipocyte differentiation stages. Significantly up-regulated TFs expressed at the different time points (12 h, 24 h, 48 h and 72 h) are shown in the top half of the panel, while significantly down-regulated transcription factors at the same time points are shown in the lower half of the panel

Activation and repression of defined transcription factors are essential for the commitment of progenitors to a specific differentiation lineage, setting the stage for a gene expression pattern characteristic of each mature cell type. Adipocyte differentiation is regulated by multiple TFs, and cooperative interactions among these transcription factors drive the expression of downstream target genes that are necessary for the generation and maintenance of adipocyte characteristics such as lipid accumulation and insulin sensitivity. A total of 164 differentially expressed TFs were obtained by alignment of DEGs with ITFP and TRANSFAC databases (Additional file 9: Table S7). A map of TF signature patterns at different time points during the differentiation process is shown in Fig. 5. Some TFs, such as E2F Transcription Factor 1 (*E2F1*), E2F Transcription Factor 5 (*E2F5*), Nuclear Receptor Subfamily 3 Group C Member 1 (*NR3C1*) and Krüppel Like Factor 5 (*KLF5*), which directly induce *PPARγ* expression and initial preadipocyte differentiation, were immediately up-regulated in the early differentiation stage (Fig. 5; Additional file 10: Figure S3). Similarly, some TFs involved in the inhibition of adipocyte differentiation are also enriched in the early negative response gene set, including GATA Binding Protein 2 (*GATA2*), GATA Binding Protein 3 (*GATA3*), HES Family BHLH Transcription Factor 1 (*HES1*) and Myogenic Differentiation 1 (*MYOD1*) (Fig. 5; Additional file 10: Figure S3). A considerable number of TFs that were not reported to be involved in regulation networks of adipocyte differentiation included Zinc Finger Protein 469 (*ZNF469*), SRY-Box 11 (*SOX11*) and Transcription Factor 3 (*TCF3*) (Fig. 5; Additional file 10: Figure S3).

### Validation of DEGs by RT-qPCR

Three samples from 0 h, 12 h, 24 h and 48 h were randomly selected for RT-qPCR to validate some key factors involved in adipose differentiation. These included *E2F1*, *E2F5*, *PPARγ*, *GSK3β*, *CCND1*, *AXIN1*, *SOX11* and *ZNF469*. The same cell samples used in mRNA-seq were used for RT-qPCR validation. The results showed that the expression patterns of these genes were highly consistent with the mRNA-seq results (Additional files 11, 12: Table S8, Figure S4).

## Discussion

This study is the first report to investigate global transcriptome changes during preadipocyte differentiation in ducks. Here, we not only obtained a relatively complete dynamic transcriptome map of subcutaneous fat differentiation in ducks, but also found many known or unknown TFs and signaling pathways associated with preadipocyte proliferation and differentiation, which is beneficial for determining optimal breeding for subcutaneous fat deposition in Pekin duck.

### Regulation events of preadipocyte proliferation

**Fig. 6:**
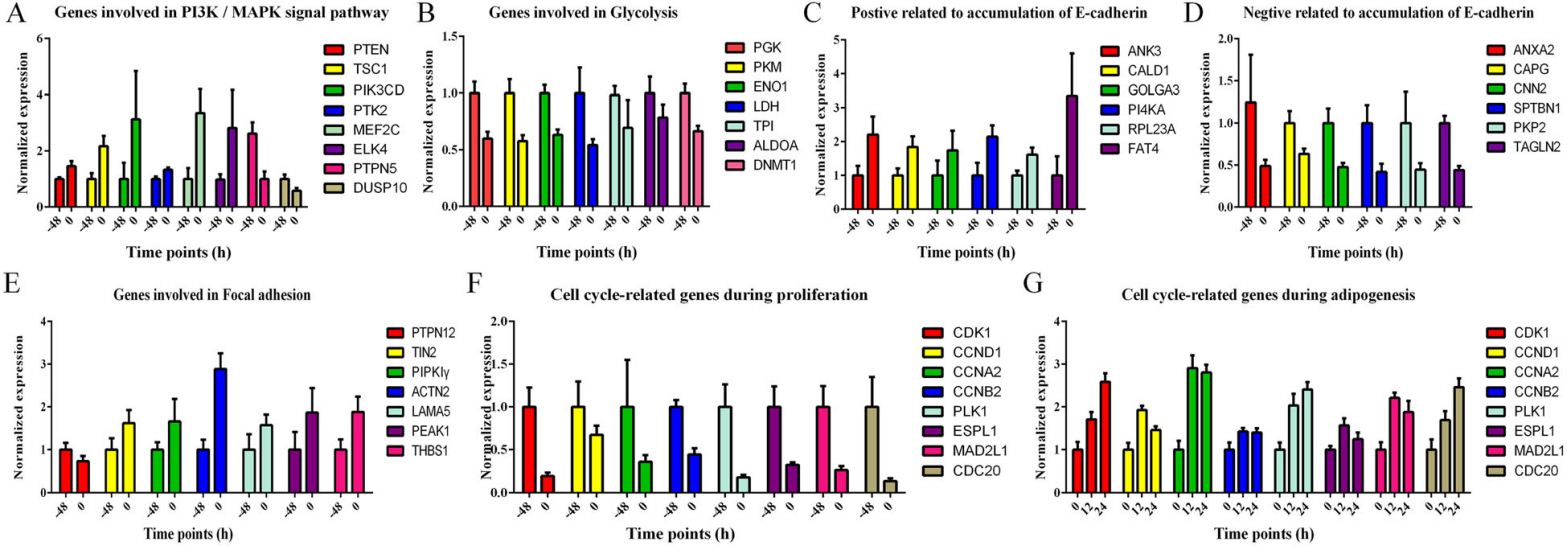
mRNA-seq expression patterns of some key regulatory or functional genes responsible for regulating pathways during proliferation or differentiation stages. (**a**) Genes involved in PI3K / MAPK signal pathway. (**b**) Genes involved in glycolysis. (**c**) Positive related to accumulation of E-cadherin. (**d**) Negative related to accumulation of E-cadherin. (**e**) Genes involved in focal adhesion. (**f**) Cell cycle related genes during proliferation stage. (**g**) Cell cycle related genes during differentiation stages

A total of 845 DEGs were obtained between -48 h and 0 h by pair wise comparison. The premise of inducing preadipocyte differentiation is that the proliferating preadipocyte become growth-arrested by contact inhibition. This process is accompanied by many regulatory events, ultimately providing a special microenvironment for initiating differentiation. Thus, GO enrichment and pathway analysis were performed to explore the functions of DEGs during proliferation. As expected, consistent with growth arrested, the cell cycle and related pathways were significantly down-regulated; for example cell cycle, DNA replication, glycolysis and PI3K-Akt signaling pathway during different proliferation stages. Meanwhile, proliferation associated with basic cellular and metabolic processes (transcription, ribosome biogenesis, translation and protein folding) were also down-regulated. The PI3K-Akt signaling pathway is a major mediator of cellular proliferation, survival and differentiation [26]. Phosphatase And Tensin Homolog (*PTEN*), a primary and classical inhibitor of the PI3K-Akt pathway [27], was significantly up-regulated at 0 h (Fig. 6a). Some previous tumor research has reported that the PI3K-Akt pathway, glycolysis and DNA Methyltransferase 1 (*DNMT1*) cooperate to activate cell proliferation and cross-regulate each other in a positive feedback loop to provide the sufficient amount of ATP and metabolic intermediates required for rapid proliferation [28, 29]. We further analyzed changes in the transcriptional levels of *DNMT1* and several genes associated with glycolysis. Interesting, the expression of these genes showed a very high agreement with the PI3K pathway, which was significantly down-regulated at 0 h (Fig. 6B). In addition, *DNMT1* is a major DNA methyltransferase responsible for maintaining self-renewal and the undifferentiated state in mesenchymal stem cells [30], whereas its knockout can accelerate preadipocyte differentiation [31], and the expression patterns of *PPARγ* and *DNMT1* showed opposite trends in some cell lines [32].

**Fig. 7:**
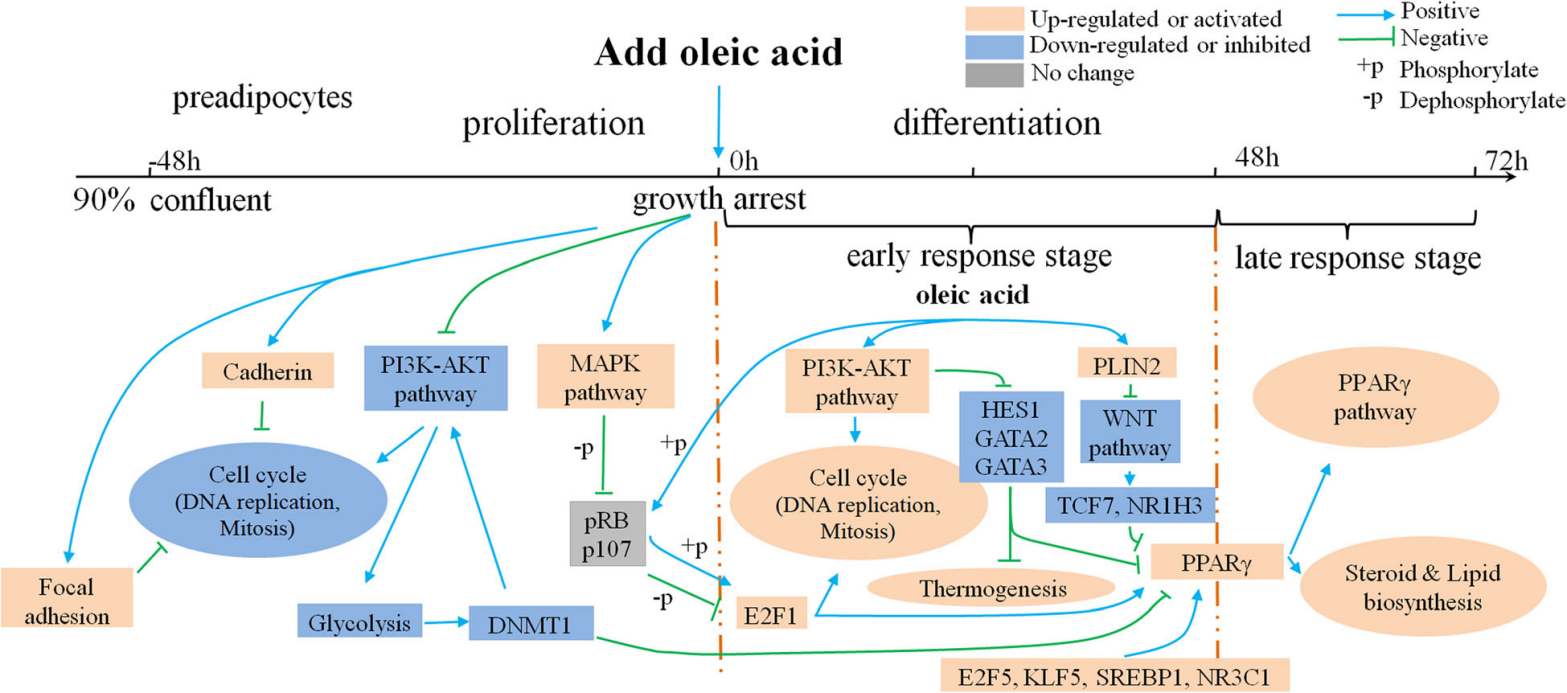
Regulation network models in duck subcutaneous preadipocyte proliferation and differentiation stage. The pink or blue boxes represent the genes or pathways, which were identified by our study, while the gray boxes represent genes from published literature

On the other hand, cadherin binding, calcium ion binding, focal adhesion and MAPK signaling pathway were up-regulated at 0 h. Cadherin is a calcium-dependent cell adhesion molecule that is important for the formation of adherens junctions to bind cells to each other, and the loss of its function can directly promote cell proliferation and tumor progression [33]. Moreover, mitotic cell cycle phase transition is inhibited by over-expression of cadherin in cells, which is down-regulated at 0 h [34, 35] (Fig. 6c, d). 26 and 22 focal adhesion terms were also significantly enriched at -48 h and 0 h respectively. Focal adhesion is the primary site of cell adhesion to the substrate, which links the extracellular matrix, via membrane-bound receptors, to the cell’s cytoskeleton, and plays a critical role in many fundamental processes such as embryonic morphogenesis, angiogenesis and inflammation [36, 37]. We observed that the expression of Protein Tyrosine Phosphatase Non-Receptor Type 12 (PTPN12), an important phosphatase which enables increased focal adhesions as well as inhibits tumor growth [38], was significantly down-regulated at 0 h. Similarly, both Talin2 (*TIN2*) and phosphatidylinositol phosphate kinase type I Gamma (*PIPKIγ*) play a role in focal adhesion formation [39], and their expression increased at 0 h (Fig. 6e). According to the above analysis, both of the cadherin binding terms and the focal adhesion terms play a negative role in cell cycle transition and mitosis of duck preadipocyte (Fig. 7).

The MAPK signaling pathway relies on a series of phosphatase cascades to sustain activation of p38α, ultimately inhibiting cell proliferation [40, 41]. Dual Specificity MAP Kinase Phosphatase 10 (*DUSP10*) or Protein Tyrosine Phosphatase, Non-Receptor Type 5 (*PTPN5*) which act as upstream inhibitors and dephosphorylase p38α, were significantly decreased, whereas Myocyte Enhancer Factor 2C (*MEF2C*) and ETS Domain Containing Protein 4 (*ELK4*), which act downstream of p38α [40, 42, 43], were increased at 0 h (Fig. 6a). In fact, the activated p38 will further dephosphorylate the Retinoblastoma (pRB) and Cyclin Dependent Kinase Inhibitor 1B (p27^Kip1^) in confluent cultures [41]. Furthermore, the dephosphorylated pRB binds to the E2F binding site on the *PPARγ* promoter, preventing E2F1 triggering the expression of *PPARγ* during the early stages of adipogenesis [44, 45]. All these results suggested that there may also be such a positive feedback loop in duck subcutaneous preadipocyte that is directly or indirectly involved in the regulation of cell proliferation and differentiation (Fig. 7).

### Regulation of preadipocyte differentiation

Differentiation itself is characterized by changes in cell morphology and regulated by complex molecular events that are initiated by adipogenic hormonal stimulus. Based on co-expression network analysis, we divided the DEGs obtained at different stages of differentiation into three response gene sets: early positive response, early negative response and late response gene sets.

Regulation of actin cytoskeleton, DNA replication, PI3K-Akt signaling pathway and other functional pathways were significantly enriched in the early positive response gene set, with most of them having been reported to be involved in the early stage of the differentiation [46–48]. DNA replication is one of the key events taking place in early adipogenesis, and inhibition of DNA synthesis at this stage blocks differentiation [49]. A hall mark of differentiation is a pronounced change in cell shape, which is determined by dynamics of the actin cytoskeleton [50]. The consequent rapid increase in actin leads to interaction of actin with other adipogenic inhibitors, and allows expression of PPARγ followed by adipogenic differentiation [51]. Interesting, many cell cycle-related genes and the PI3K-Akt signaling pathway, which were down-regulated during the proliferation stage, increased significantly during the differentiation stage (Fig. 6F, G). This is not surprising as those contact inhibited preadipocyte re-enter the cell cycle after hormonal induction, arrest proliferation and, again, finally undergo terminal differentiation [6]. More importantly, the inducing agent, oleic acid, has been reported to stimulate the proliferation of various cells by activating the PI3K-Akt pathway [52, 53]. Phosphorylated pRB combined with highly expressed *CCND1* releases activated E2F1, ultimately initiating *PPARγ* transcription [44]. Moreover, *E2F1* global knockout mice have a limited ability to accumulate adipose tissue in response to high-fat feeding [44]. Corroborating this, some mice enable developing adipose depot act on *E2F1* to stimulate *PPARγ* without the need for other early adipogenesis transcription factors, such as CCAAT Enhancer Binding protein β or δ (*C/EBPβ*, *C/EBPδ*) [8, 54]. These reports and our transcriptomic profiling together suggests that, oleic acid- PI3K-Akt pathway- pRB- E2F1 -mediated activation cascade mechanism could facilitate the expression of *PPARγ* during the early stage of differentiation (Fig. 7).

As expected, *WNT*, *HIF*, *FOXO* and other genes known to be involved in the inhibition of the preadipocyte differentiation signaling pathway, were significantly enriched in the early negative response gene set. The WNT signaling pathway maintains preadipocyte in an undifferentiated state through inhibition of *C/EBPα* and *PPARγ*. The canonical WNT signaling pathway inhibits the kinase activity of complexes containing glycogen synthase kinase 3 β (GSK3β), Axis Inhibitor 1 (Axin1), β-catenin and other proteins [55]. This complex targets β-catenin for rapid degradation through phosphorylation [56]. Thus, once hypophosphorylated due to WNT signaling, β-catenin is stabilized and translocates to the nucleus where it binds the TCF/LEF family of transcription factors to negatively regulate *PPARγ* transcription [56, 57]. Consistent with this, expression of *PLIN2*, *GSK-3β* and *AXIN1* increased rapidly at 12 h, while expression of TCF/LEF family transcription factor, T Cell Specific Transcription Factor 7 (*TCF7*) and Nuclear Receptor Subfamily 1 Group H Member 3 (*NR1H3*) decreased significantly. On the other hand, oleic acid has been reported to stimulate the expression of Perilipin2 (*PLIN2*) in 3 T3-L1 cells [58]. Meanwhile, *PLIN2* has been shown to activate *AXIN1* and *GSK3β*, thereby inhibiting the WNT signaling pathway [59]. Consistent with these reports, our data predict that an oleic acid- PLIN2- WNT pathway- β-catenin- TCF7 mediated negative regulatory cascade mechanism could further enhance the expression of *PPARγ* during the early stage of differentiation (Fig. 7).

In addition, we found more differentially expressed TFs at the early response stage (0 h–48 h) compared to late response stage (48 h–72 h). *NR3C1* [60], *KLF5* [61] and Sterol Regulatory Element Binding Transcription Factor 1 (*SREBP1*) [62], which directly or indirectly facilitate the expression of *PPARγ*, were significantly up-regulated within 24 h (Additional file 10: Figure S3). At the same time, some TFs known to inhibit adipocyte differentiation also decreased significantly, including *GATA2* [63], *GATA3* [63, 64], *HES1* [65] and *MYOD1* [66] (Additional file 10: Figure S3), with most of them having been reported to be inhibited by the activated PI3K-AKT signaling pathway. Although the brown adipose differentiation pathway associated with thermogenicity has been lost in the avian lineage [67], we also found several brown adipose determination TFs increased significantly at the early response stage. These include PPARG Coactivator 1 Alpha (*PGC1α*) [68], Euchromatic Histone Lysine Methyltransferase 1 (*EHMT1*) [69] and PR/SET Domain 16 (*PRDM16*) [70] (Additional file 10: Figure S3). In fact, the formation of brown adipose tissue shares many common differentiation regulatory nodes with white adipose tissue [71], so their rise may be related to the decline of differentiation inhibitors, such as *GATA2*, *GATA3* and *HES1* (Fig. 7). However the role of these genes in duck subcutaneous preadipocyte differentiation requires further investigation.

The lipid biosynthetic process, steroid biosynthetic process, PPAR*γ* and p53 signaling pathways implicated in the regulation of lipid and lipoprotein metabolism [72, 73], were enriched in the late response stage. At the same time, some of the markers related to fat metabolism and nutrient transport, which were also highly expressed in the late stage of preadipocyte differentiation, also increased significantly, with the expression of particular genes even increasing by more than 50 fold (i.e., *FABP4*, *PLIN2*). This is consistent with previous studies which show that the preadipocyte have been transformed into adipocyte after 72 h of induction [19].

Finally, we also identified TFs (i.e., *ZNF469* and *SOX11*) that have not been reported previously to be involved in the regulation of adipose differentiation, which were up-regulated during the differentiation stage. *ZNF469* has been proposed as a candidate gene for keratoconus, and its mutation is associated with brittle cornea syndrome [74]. Previous study on the evolution of adipose tissue has shown that the *ZNF469* gene in Pekin duck is highly variable compared with its wild ancestor, which may be one of the factors causing the excessive deposition of adipose tissue in Pekin duck [75]. *SOX11* was reported to inhibit osteogenic differentiation of preadipocyte, but the relationship with adipocyte differentiation has not been reported [76]. In fact, preadipocyte are delicately balanced for their differentiation direction - numerous in vitro investigations have demonstrated that adipose-induction factors inhibit osteogenesis, and conversely, bone-induction factors hinder adipogenesis [66]. Taken together, we speculate that the *ZNF469* and *SOX11* have a positive effect on Pekin duck subcutaneous preadipocyte differentiation.

### Genes with high expression across all stages

Dynamic changes in gene expression reflect intrinsic mechanisms of an organism’s response to developmental and environmental signals. Although genes with high and constant expression levels in all stages may also exhibit the characteristics of the cell itself. In the present study, 1000 genes with FPKM over 30 (Additional file 13: Table S9) were selected to carry out functional enrichment analysis. As expected, transcription, ribosome biogenesis, translation and protein folding were identified, indicating active growth and metabolism in adipose cells and tissues [77]. Extracellular matrix (ECM)-receptor interaction was also significantly enriched in these genes (Additional file 14: Table S10). The ECM of adipose tissues undergoes constant remodeling to allow adipocytes and their precursor cells (preadipocytes) to change cell shape and function in adaptation to nutritional cues by interacting with the receptor on the cell surface [78]. Moreover, as belonging to the ‘receptor’ term integrins subunit beta 1 (*ITGB1*) and *CD44* were positively correlated with insulin resistance and glycemic control in human subjects [79, 80]. In line with this, HFD-fed CD44 knockout mice remained considerably more insulin-sensitive and glucose- tolerant than HFD-fed wild-type control mice and exhibited lower blood insulin levels [81]. Furthermore, domestic poultry adipose tissue is considered to be fairly insensitive to insulin (insulin resistance) with lipolysis being under glucagon control, due to intensive genetic selection for rapid growth [82]. In this sense, these highly expressed ECM receptors may contribute to insulin resistance in poultry.

## Conclusions

This study is the first report exploring transcriptome changes during the differentiation of preadipocyte into adipocyte in ducks. In total, 845 and 3382 DEGs were identified in the preadipocyte proliferation and differentiation stages. We not only found many known and novel TFs and signaling pathways associated with duck preadipocyte proliferation and differentiation, but also provide a proposed regulation network model of subcutaneous preadipocyte differentiation. Our study provides a solid transcriptional analysis with which to facilitate functional studies on preadipocyte differentiation in ducks.

## Methods

### Duck subcutaneous preadipocytes isolation

Pekin ducks were provided by Beijing Golden Star Ltd. All ducks in this study were given continuous access to a standard commercial feed ration and water as described in our previous study [4, 21]. In order to reduce the suffering of animals, three ducks were moved to the laboratory which provided isolation, thereby minimizing noise and distractions. Ducks were sacrificed under deep anesthesia with sodium pentobarbital (Sigma). The subcutaneous adipose tissues were collected for the primary culture of subcutaneous preadipocytes. The experimental procedure was in accordance with the guidelines of the China agricultural University Animal Care Committee. Subcutaneous preadipocytes from three ducks were prepared by the method as described before [19], with some modifications. Briefly, subcutaneous adipose tissue was collected under sterile conditions from a 16-day-old female duck and washed with PBS. The clean adipose tissue was minced into fine sections and digested with 15 mL of digestion Solution [DMEM/F12 (Dulbecco’s modified Eagle’s medium/Ham’s nutrient mixture F-12), 100 mM HEPES, 4% BSA, 2 mg/mL collagenase I (Invitrogen), pH 7. 4] for 65 min at 37 °C in a water bath shaker. After incubation, growth medium (DMEM/F12, 10% FBS, 100 U/mL penicillin and streptomycin) was added to stop digestion. The mixture was filtered through nylon screens with 70 μm mesh openings to remove undigested tissue and large cell aggregates. The filtered suspensions were centrifuged at 300×g for 10 min to separate floating adipocytes from preadipocytes. The harvested preadipocytes were then re-suspended with 10 mL of Blood Cell Lysis Buffer (Invitrogen), and incubated at room temperature for 10 min. Finally, the obtained preadipocytes were seeded into T25 flasks at a suitable density and cultured in a humidified atmosphere of 95% air and 5% CO_2_ at 37 °C until 90% confluency. The preadipocytes were then serially subcultured at a 1:2 split ratio until differentiation experiments began.

### Induction of duck preadipocytes differentiation

Prepared duck preadipocytes were seeded into 6-well plates at a density of 1 × 10^5^ cells per well and cultured with growth medium until achieving 90% confluence. After 2 days, the growth medium was removed and replaced with differentiation medium (growth medium supplemented with 300 μM oleic acid) and medium was changed every 2 days until day 3 of differentiation, which was similar to the procedure used with chicken preadipocytes [83]. The design and sampling strategy are described in Additional file 15: Figure S5. Cells were collected for mRNA-seq at -48 h, 0 h, 12 h, 24 h, 48 h and 72 h. Each interval included six biological replicates (*n* = 6), with 36 samples collected for mRNA-seq in total.

### Oil red O staining and measurement of lipid droplet accumulation

Lipid droplets were stained with oil red O (Sigma) according to Shang Z et al. [83]. Briefly, the cells were washed three times with PBS and fixed with 10% (v/v) paraformaldehyde for 30 min at room temperature. Then cells were washed with PBS and stained with 1% Oil Red O working solution [Oil Red O dye in 60% (v/v) isopropyl alcohol] for 40 min. The cells were counterstained with Hoechst 33342 after removing the residual Oil Red O and repeatedly washed using distilled water. Staining work at each time point included three biological replicates (*n* = 3). Finally, observation and photographing of cell phenotypes were conducted under an inverted fluorescent microscope (Leica) at 200X magnification.

Lipid droplet accumulation was measured by oil red O extraction assay. First, oil red O stained cells were prepared by the above method. Then, oil red O was extracted by adding 1 mL of 100% (v/v) isopropyl alcohol, and measured at 500 nm using an ultraviolet spectrophotometer (Pharmacia). Adjacent plate wells with identical treatment were trypsinized, diluted and counted with a hemocytometer to normalize the extraction results [83, 84].

### Glycerol-3-phosphate dehydrogenase (GPDH) assay

GPDH is a rate-limiting enzyme for fatty acyl-CoA biosynthesis and its enzyme activity will rise significantly in the late stages of differentiation. The differentiated preadipocytes were collected at 0 h, 48 h, and 96 h. GPDH assay was conducted using a GPDH Activity Colorimetric assay kit (Sigma). Each time point included three biological replicates for GPDH analysis (n = 3). Protein concentrations of cell culture homogenates were determined by BCA protein assay kit (Sigma) using bovine serum albumin as the standard. GPDH activity was reported as nmol/min/mL [19].

### RNA extraction and cDNA library preparation

The different stages of clean preadipocytes were homogenized in TRIzol (Invitrogen) and processed following the manufacturer’s protocol. The quantity and quality of RNA were assessed via Nanodrop. All RNA samples had an RNA integrity number value > 8.0, and an optical density 260:280 ratio > 1.9. Approximately 5 μg of total RNA was then used for mRNA-seq using the Illumina sequencing platform. Briefly, the mRNA was enriched using magnetic beads with oligo (dT) primer, and then randomly fragmented using Fragmentation buffer. The first-strand and the second-strand cDNA were synthesized using First Strand Enzyme Mix and Second Strand/End Repair Enzyme Mix (Vazyme Biotech). The products were purified by AMPure XP beads (Beckman Coulter) and the end of the double strand was then repaired and A-tailed. Suitably sized fragments were selected using AMPure XP beads (Beckman Coulter) to construct the cDNA library by PCR. Following construction, double-stranded cDNA libraries were sequenced on an Illumina HiSeq X-10 with PE150 mode at the Novogene Inc.

### Assembly-guided transcript discovery

The raw reads with adaptors removed were filtered according to the following criteria: 1) reads with unknown nucleotides (N) larger than 5%; 2) reads containing more than 30% bases with Q-value < 20. The clean reads were used for further analysis.

The mRNA-seq guide-assembly was performed using the HISAT2 and StringTie pipeline [85]. The paired-end reads of adipose samples were aligned to the duck reference genome individually using the hierarchical indexing for spliced alignment of transcripts program HISAT2 (−v2.0.5). For this purpose, we built an index file for the duck reference genome (*Anas_platyrhynchos*.BGI_duck_1.0) using HISAT2-build. StringTie was used to assemble each sample based on alignment file for each sample and merged all predicted transcripts into a unified transcript model. We compared reference-guided transcripts with the known annotations to assess the quality of transcript predictions.

The novel transcripts from assembled transcripts were extracted using gffread and annotated with known protein sequences database from Uniprot (www.uniprot.org) using the Blastx algorithm with a cutoff e-value of 10^− 5^. Ultimately, we merged all known transcripts and new annotated transcripts into a non-redundant gene set for quantification and differential expression analysis.

### Differentially expressed gene identification

Transcripts were quantified via the Salmon (−v0.8.2) software using the transcriptome-based quasi-mapping mode, and clean reads of samples were mapped to the gene set individually. Once expression level for each transcript in each sample (− 48 h, 0 h, 12 h, 24 h, 48 h and 72 h) was quantified, the data were summarized to a gene-level. First, we calculated sample-to-sample distances to assess the data quality using DESeq2 (version1.16.1) [86]. Differential expression analysis at gene-level between 6 time points of adipose samples was performed using DESeq2. Significance for differential expression was accepted at the Benjamini-Hochberg adjusted P (FDR) < 0.05 level, and fold change (FC) > 1.5. Finally, we used Metascape (http://metascape.org) to get the enriched GO terms and KEGG pathways of differentially expressed genes (DEGs). In order to avoid poorly expressed genes, genes with FPKM < 0.5 were filtered before conducting DEG analysis.

### Construction and visualization of co-expression network

The weighted correlation network analysis (WGCNA) relies on the hypothesis that strongly correlated expression levels of a group of genes, referred to as “modules”, may work cooperatively in related pathways, contributing together to the phenotype [87]. We found clusters (modules) of highly correlated DEGs, for summarizing such clusters using the module eigengene or an intramodular hub gene, for relating modules to one another (using eigengene network methodology), and for calculating module membership measures using WGCNA. In order to analyze the influence of power value on the scale independence and mean connectivity, we used the function connectivity from package WGCNA, with the “randomly selected genes” parameter set at 4000, other parameters set as default, and the power parameter pre-calculated by the pickSoft Threshold function of WGCNA. We next summarized the expression values using the function collapse Rows implemented in the R package WGCNA. The interactions (correlations) of each module were analyzed and visualized by heat map. Further, the co-expression network of highly coordinated genes among most of the modules was visualized and analyzed by Cytoscape (version 2.8.3).

### GO and KEGG pathway enrichment analyses

To investigate genes from one gene ontology GO term (http://metascape.org/gp/index.html), a hypergeometric *p*-value was calculated and adjusted as a q-value, where the background was set to be genes in the whole genome. GO terms with q < 0.05 were considered significantly enriched, and GO enrichment analysis elucidated the biological functions of the DEGs. The log10 value (p-value) denotes enrichment scores that represent the significance of GO term enrichment among DEGs. Kyoto Encyclopedia of Genes and Genomes (KEGG) pathway analysis was also performed to predict the molecular interactions and reaction networks associated with DEGs. Using the same method as that used for GO enrichment analysis, significantly enriched KEGG pathways were identified.

### Identification of transcription factors from DEGs

ITFP (http://itfp.biosino.org/itfp) and TRANSFAC (http://www.gene-re gulation.com/pub/databases.html) provide data on eukaryotic transcription factors. Data relating to human transcription factors, and their binding site motifs were downloaded from ITFP and TRANSFAC. Based on the downloaded data, DEGs were used for screening transcription factors. Furthermore, we assembled a time-specific map of the expression of transcription factors after induction based on DEGs obtained during the differentiation stage. The selected and displayed differentially expressed TFs from different time points were identified using a t-test at a fold change of ≥1.5 and probability (*P* < 0.05) compared with 0 h.

### Validation of mRNA-seq data using quantitative real-time PCR

We randomly selected three samples from 0 h, 12 h, 24 h and 48 h, which were consistent with the library preparation sample, for RT-qPCR and calculated their correlation with the corresponding FPKM in mRNA-seq data. First strand cDNA was synthesized using the PrimeScript RT Master Mix kit according to the supplier’s protocol (Takara Bio Inc). Pairs of primers for each gene were designed from the CDs sequence of the target gene from the National Centre for Biotechnology Information (NCBI) (Additional file 11: Table S8). Quantitative real-time (RT-qPCR) was performed in duplicate reactions including SYBR Premix ExTaq II (Takara Bio Inc), specific forward and reverse primer, diluted cDNA and RNase free water. Quantification of selected gene expression was performed using the comparative threshold cycle (2^-ΔΔCT^) method by normalizing the expression of the target genes to a reference gene (*GAPDH*). The RT-qPCR results for all genes were statistically tested using the Student’s t-test.

### Generation of gene network

We conducted further analysis of the DEGs obtained in our study and manually assembled a proposed regulation network model of preadipocyte proliferation and differentiation based on published literature (Fig. 7). Previous study reported that Cadherin, focal adhesion, DNA methylation and PI3K-AKT signaling pathway are mainly involved in regulation of cell cycle, mitosis and cancer. Similarly, several studies have revealed the importance of some TFs and pathways in regulating adipogenesis both in vitro and in vivo*.* These include *PPARγ* [6], *E2F1* [44], *GATA2* [63], PI3K-AKT [26, 53] and WNT signaling pathway [57]. Also, in our main network analysis, these TFs and pathways, were affected by growth arrest and oleic acid, consistent with their role in proliferation and differentiation, and were therefore incorporated from regulation network analysis of duck subcutaneous preadipocyte differentiation.

## Additional files

Additional file 1: **Table S1.** Statistics relating to the mRNA-seq data. (XLSX 12 kb)

Additional file 2: **Figure S1.** Heatmap of the differentiation of biological replicates of duck subcutaneous preadipocyte. The colors ranging from white to blue represent Pearson correlation coefficients ranging from 0 to 1, indicating low to high correlations, respectively. (PDF 498 kb)

Additional file 3: **Table S2.** List of genes expressed during the entire stage. (XLSX 8936 kb)

Additional file 4: **Table S3.** List of all DEGs during complete duck subcutaneous preadipocyte differentiation. (XLSX 929 kb)

Additional file 5: **Table S4.** GO term analysis of DEGs at different stages. (XLSX 248 kb)

Additional file 6: **Table S5.** KEGG pathway analysis of DEGs at different stages. (XLSX 51 kb)

Additional file 7: **Table S6.** Genes involved in eight modules. (XLSX 147 kb)

Additional file 8: **Figure S2.** Classification of DEGs throughout the entire differentiation process and visualization of gene expression levels of significant modules. (PDF 1241 kb)

Additional file 9: **Table S7.** List of differentially expressed TFs obtained in differentiation stage. (XLSX 30 kb)

Additional file 10: **Figure S3.** mRNA-seq expression patterns of some key functional genes or TFs during differentiation stage. (PDF 629 kb)

Additional file 11: **Table S8.** Validation of mRNA-seq using RT-qPCR. (XLSX 28 kb)

Additional file 12: **Figure S4.** Validation of some key regulatory factors during adipocyte differentiation stage (0 h, 12 h, 24 h and 48 h) using RT-qPCR. Transcript abundance is presented as fold change± S.E.M (*n* = 3) using GAPDH as a reference gene (delta-delta method). (PDF 387 kb)

Additional file 13: **Table S9.** List of selected genes with FPKM >30 and stably expressed during the entire stage. (XLSX 582 kb)

Additional file 14: **Table S10.** GO terms and KEGG pathway analysis of selected genes with FPKM >30 and stably expressed during the entire stage. (XLSX 80 kb)

Additional file 15: **Figure S5.** Design and sampling strategy of the differentiation process of duck subcutaneous preadipocyte. (PDF 1095 kb)

## Data Availability

The data supporting the conclusions of this article (raw mRNA-seq reads) are available in the National Center for Biotechnology Information (NCBI) Sequence Read Archive (SRA) under accession number SRX4646736 (https://www.ncbi.nlm.nih.gov/sra/SRX4646736).

## Abbreviations

*AXIN1*: Axis Inhibitor 1
BAT: Brown adipose tissue
*C/EBPα*: CCAAT Enhancer Binding Protein Alpha
*C/EBPδ*: CCAAT Enhancer Binding Protein Delta
*CCND1*: G1/S Specific CyclinD1
DEGs: Differentially expressed genes
*DNMT1*: DNA Methyltransferase 1
*DUSP10*: Dual Specificity MAP Kinase Phosphatase 10
*E2F1*: E2F Transcription Factor 1
*E2F5*: E2F Transcription Factor 5
*EHMT1*: Euchromatic Histone Lysine Methyltransferase 1
*ELK4*: ETS Domain Containing Protein 4
*FABP4*: Fatty Acid Binding Protein 4
*GATA2*: GATA Binding Protein 2
*GATA3*: GATA Binding Protein 3
GO: Gene ontology
*GPDH*: Glycerol‑3‑phosphate dehydrogenase
*GSK3β*: Glycogen Synthase Kinase 3 Beta
*HES1*: Hes Family BHLH Transcription Factor 1
*ITGB1*: Integrins Subunit Beta 1
KEGG: Kyoto encyclopedia of genes and genomes
*KLF5*: Kruppel Like Factor 5
*MEF2C*: Myocyte Enhancer Factor 2C
*MYOD1*: Myogenic Differentiation 1
NCBI: National Center for Biotechnology Information
*NR1H3*: Nuclear Receptor Subfamily 1 Group H Member 3
*NR3C1*: Nuclear Receptor Subfamily 3 Group C Member 1
*p27Kip1*: Cyclin Dependent Kinase Inhibitor 1B
*PGC1α*: Phosphatidylinositol phosphate kinase type I Gamma
*PIPKIγ*: PPARG Coactivator 1 Alpha
*PLIN2*: Perilipin2
*PPARγ*: Peroxisome Proliferator Activated Receptor Gamma
*PRDM16*: PR/SET Domain 16
*PTEN*: Phosphatase And Tensin Homolog
*PTPN12*: Protein Tyrosine Phosphatase Non-Receptor Type 1
*PTPN5*: Protein Tyrosine Phosphatase, Non-Receptor Type 5
*RB*: Retinoblastoma
RT-qPCR: Quantitative real-time Polymerase Chain Reaction
*SCD1*: Stearoyl CoA Desaturase 1
*SOX11*: SRY-Box 11
*SREBP1*: Sterol Regulatory Element Binding Transcription Factor 1
*TCF3*: Transcription Factor 3
*TCF4*: T Cell Specific Transcription Factor 4
*TCF7*: T Cell Specific Transcription Factor 7
TFs: Transcription factors
*TIN*: Talin
*UCP1*: Uncoupling Protein 1
*ZNF469*: Zinc Finger Protein 469
