# Supplementary figures and images for: Dynamics of transcriptome changes during subcutaneous preadipocyte differentiation in ducks
Source: BMC Genomics. 2019 Sep 2;20:688. doi: 10.1186/s12864-019-6055-9 (PMC6720933; doi:10.1186/s12864-019-6055-9)

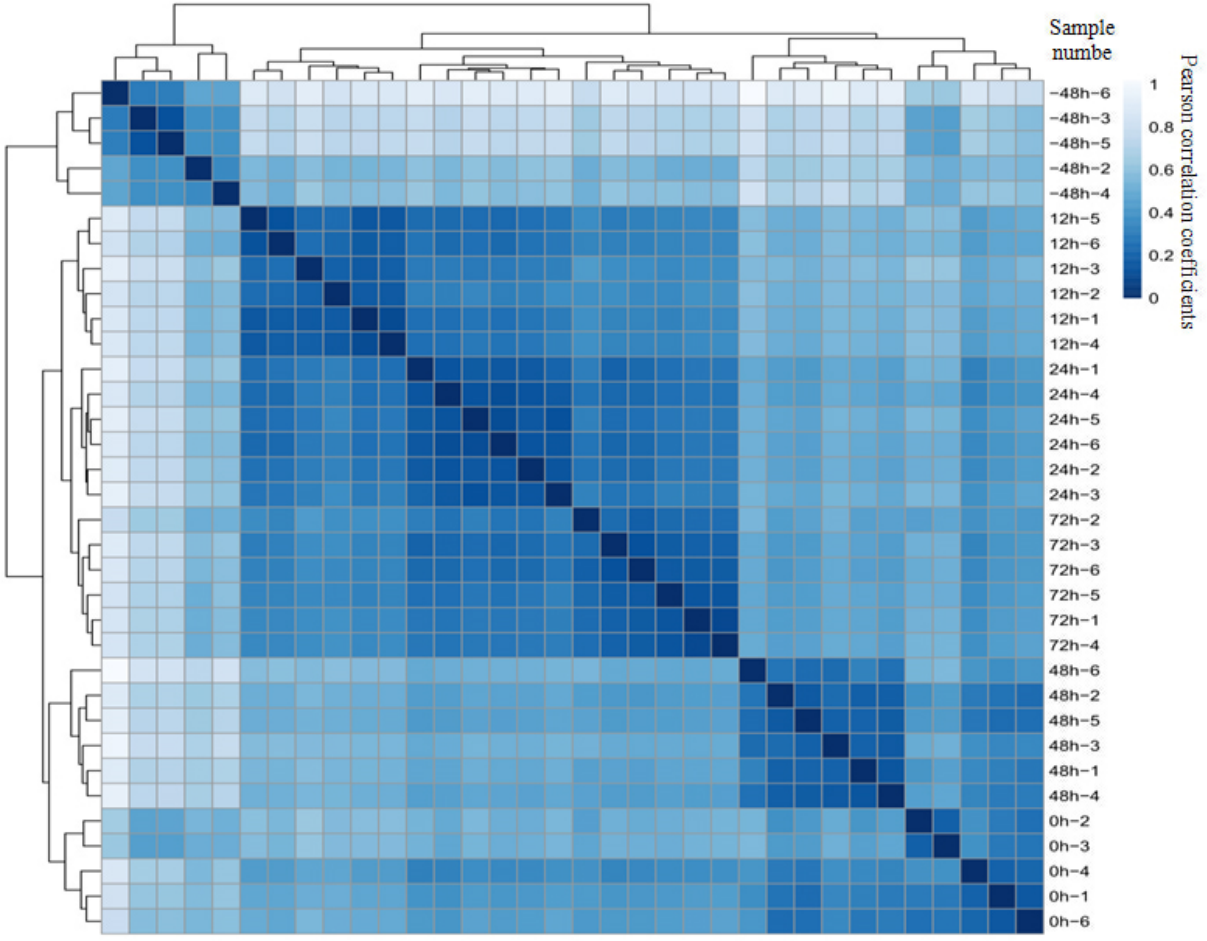

Supplement: Supplementary file 2 — Figure S1. Heatmap of the differentiation of biological replicates of duck subcutaneous preadipocyte. The colors ranging from white to blue represent Pearson correlation coefficients ranging from 0 to 1, indicating low to high correlations, respectively. (PDF 498 kb) [file 12864_2019_6055_MOESM2_ESM.pdf]

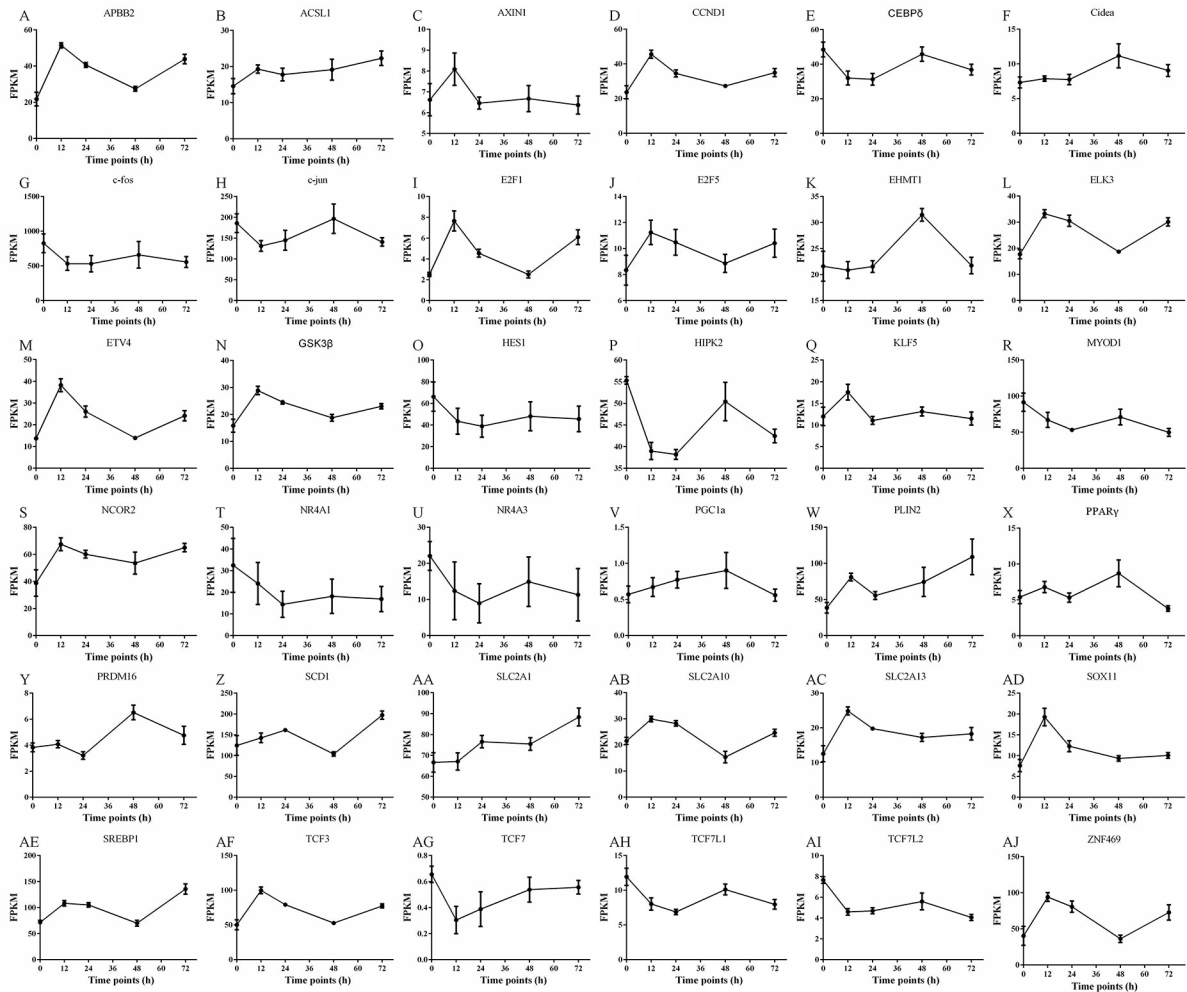

Supplement: Supplementary file 8 — Figure S2. Classification of DEGs throughout the entire differentiation process and visualization of gene expression levels of significant modules. (PDF 1241 kb) [file 12864_2019_6055_MOESM8_ESM.pdf]

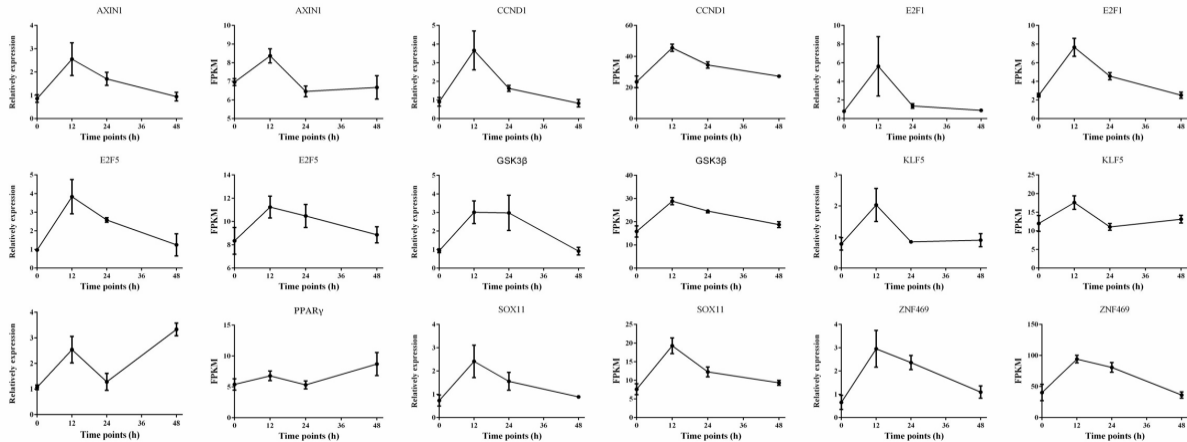

Supplement: Supplementary file 10 — Figure S3. mRNA-seq expression patterns of some key functional genes or TFs during differentiation stage. (PDF 629 kb) [file 12864_2019_6055_MOESM10_ESM.pdf]

Proliferation  
stage

Differentiation  
stage

Up-regulated

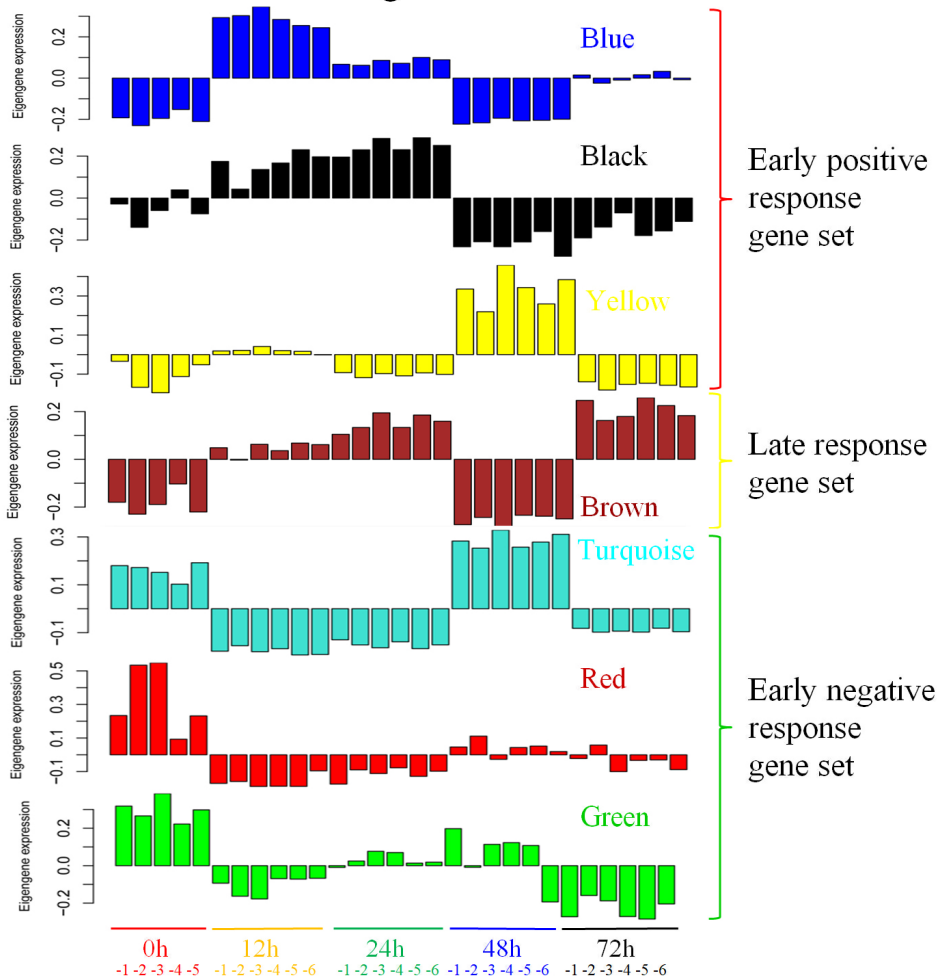

-48h

0h

Down-regulated

Supplement: Supplementary file 15 — Figure S5. Design and sampling strategy of the differentiation process of duck subcutaneous preadipocyte. (PDF 1095 kb) [file 12864_2019_6055_MOESM15_ESM.pdf]
