# Supplementary material for: Dynamics of transcriptome changes during subcutaneous preadipocyte differentiation in ducks
Source: BMC Genomics. 2019 Sep 2;20:688. doi: 10.1186/s12864-019-6055-9 (PMC6720933; doi:10.1186/s12864-019-6055-9)
Supplement: Supplementary file 12 — Figure S4. Validation of some key regulatory factors during adipocyte differentiation stage (0 h, 12 h, 24 h and 48 h) using RT-qPCR. Transcript abundance is presented as fold change± S.E.M (n = 3) using GAPDH as a reference gene (delta-delta method). (PDF 387 kb) [file 12864_2019_6055_MOESM12_ESM.pdf]

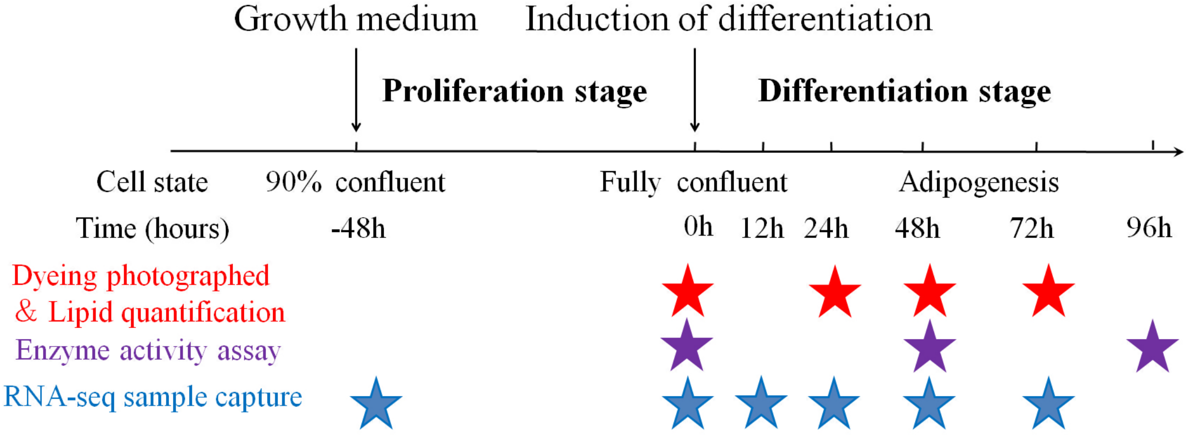

Growth medium
Induction of differentiation
Proliferation stage
Differentiation stage
Cell state
90% confluent
Fully confluent
Adipogenesis
Time (hours)
-48h
0h
12h
24h
48h
72h
96h
Dyeing photographed
& Lipid quantification
Enzyme activity assay
RNA-seq sample capture
